# Remote Acoustic Monitoring of North Atlantic Right Whales (*Eubalaena glacialis*) Reveals Seasonal and Diel Variations in Acoustic Behavior

**DOI:** 10.1371/journal.pone.0091367

**Published:** 2014-03-19

**Authors:** Leanna P. Matthews, Jessica A. McCordic, Susan E. Parks

**Affiliations:** Department of Biology, Syracuse University, Syracuse, New York, United States of America; Virginia Commonwealth Univ, United States of America

## Abstract

Remote acoustic monitoring is a non-invasive tool that can be used to study the distribution, behavior, and habitat use of sound-producing species. The North Atlantic right whale (*Eubalaena glacialis*) is an endangered baleen whale species that produces a variety of stereotyped acoustic signals. One of these signals, the “gunshot” sound, has only been recorded from adult male North Atlantic right whales and is thought to function for reproduction, either as reproductive advertisement for females or as an agonistic signal toward other males. This study uses remote acoustic monitoring to analyze the presence of gunshots over a two-year period at two sites on the Scotian Shelf to determine if there is evidence that North Atlantic right whales may use these locations for breeding activities. Seasonal analyses at both locations indicate that gunshot sound production is highly seasonal, with an increase in the autumn. One site, Roseway West, had significantly more gunshot sounds overall and exhibited a clear diel trend in production of these signals at night. The other site, Emerald South, also showed a seasonal increase in gunshot production during the autumn, but did not show any significant diel trend. This difference in gunshot signal production at the two sites indicates variation either in the number or the behavior of whales at each location. The timing of the observed seasonal increase in gunshot sound production is consistent with the current understanding of the right whale breeding season, and our results demonstrate that detection of gunshots with remote acoustic monitoring can be a reliable way to track shifts in distribution and changes in acoustic behavior including possible mating activities.

## Introduction

Understanding patterns of animal communication can reveal critical information about life history [1]. Many species rely on particular signals to locate and select mates, a process essential to individual fitness and survival of the species. In marine systems, sound is the most efficient modality for long-range communication, and acoustic signaling is the predominant form of communication for most marine species [2]. The production of loud acoustic signals for long-range communication provides an opportunity for using remote acoustic sensing to capture annual trends and seasonal changes in signal production of a variety of species [3]–[8].

Remote acoustic sensing, commonly referred to as passive acoustic monitoring (PAM), uses autonomous recorders to collect data for long periods of time with minimal disturbance to the environment. Such recording units allow researchers to collect acoustic data in remote locations or during weather conditions or seasons that would otherwise prohibit direct observation [9], [10].

In both terrestrial and marine systems, PAM has been used to investigate various aspects of habitat use and behavior by sound-producing species. For instance, passive acoustics has been used to investigate spatial and temporal dynamics of Mexican antthrush (*Formicarius moniliger*) [11] and assess the density and composition of orthopteran species assemblages [12]. Acoustic monitoring can also be used to detect species-specific signals, thereby using these signals as reliable indicators of the presence of a species within acoustic range of the receiver. For example, passive acoustics has been used to investigate the presence, behavior, and population structure of African elephant (*Loxodonta africana*) populations [13]. It has also been used in aquatic environments to track annual distribution of North Atlantic right whales (*Eubalaena glacialis*) [14], document seasonal and geographic variation in fin whale (*Balaenoptera physalus*) song [15], and monitor reproductive behavior of teleost fishes [16]. Along with presence-absence information, context-specific vocalizations enable observers to make inferences about behavior and use of a particular habitat. If the production of vocalizations associated with mating behavior shows clear seasonal trends, this can be used to identify the timing of the breeding season and the breeding locations for different species, including baleen whales [3], [4], [17]. Detection of signals with known behavioral functions can be particularly informative for identifying critical habitat areas or seasons for the protection of endangered or threatened species.

For over a decade, the critically endangered North Atlantic right whale (*Eubalaena glacialis*) has been monitored using autonomous recorders (for reviews, see [18], [19]). The right whale produces a species-specific stereotyped contact call, known as an “upcall” [20], [21]. This call has been recorded from both sexes and from all age classes, and it is therefore a reliable signal to detect the presence of right whales in an area. This is the primary call used for PAM detection studies of right whales [20], [22], [23]. Using detections of upcalls, previous studies [5] confirmed the presence of right whales in two sites on the Scotian Shelf during the late summer and early autumn. This study and others using upcalls have revealed the presence of right whales in locations or seasons otherwise overlooked from traditional visual surveys. However, the broad usage of the upcall by all individuals in the species limits the behavioral information that can be obtained from detection of this signal alone. Previous preliminary studies have indicated significant seasonal changes in right whale usage of different call types [18].

Along with tonal upcalls, right whales also produce several other stereotyped acoustic signals [20], [24], [25]. Among these is a high-intensity (>185 dB re 1μPa p-p), broadband sound known as a “gunshot” [22], [25]. The gunshot sound is a short-duration signal (<0.02 s) with a peak frequency near 1.19 kHz and a known frequency range of 20 Hz to 20 kHz, with the upper range limited by the sampling rate of the recording equipment [22], [25]. In the North Atlantic right whale, extended bouts of gunshot sound production have only been recorded from adult males on the feeding grounds, suggesting that it may function as a reproductive advertisement for females or an agonistic signal toward other males [25], [26]. Previous studies have reported recordings of individual gunshot sounds produced by nursing females in Southern right whales (*Eubalaeana australis*) [20]. It is possible that female North Atlantic right whales are also capable of gunshot sound production. However, the long stereotyped sequences of gunshot sounds observed from males have not been described in any female for any right whale species.

Male right whales also produce gunshots during surface active groups (SAGs), right whale aggregations that typically involve males competing for access to a focal female [22], [27]. Although SAGs can form in the absence of reproductively mature animals [28], the production of gunshots by adult males in SAGs with potentially reproductive compositions strengthens the association of the gunshot call with mating behavior [22].

Previous studies have detected gunshots using passive acoustic recorders during the spring, summer, and autumn months, with a marked increase in the rate of signal detection later in the season [18], [26]. Estimates of the gestation length, seasonal shifts in vocal behavior, and hormone analysis all indicate a probable right whale breeding season beginning in the autumn and lasting through the winter [18], [29]–[31]. These prior studies suggest that long-term recordings may clarify patterns of seasonality in the gunshot call and its possible association with the breeding season in right whales. In this study, we analyzed the presence of gunshots over a two-year period at two sites on the Scotian Shelf to determine whether there is any evidence that North Atlantic right whales use these habitat areas for mating-related activities.

## Materials and Methods

### Data Collection

**Figure 1:**
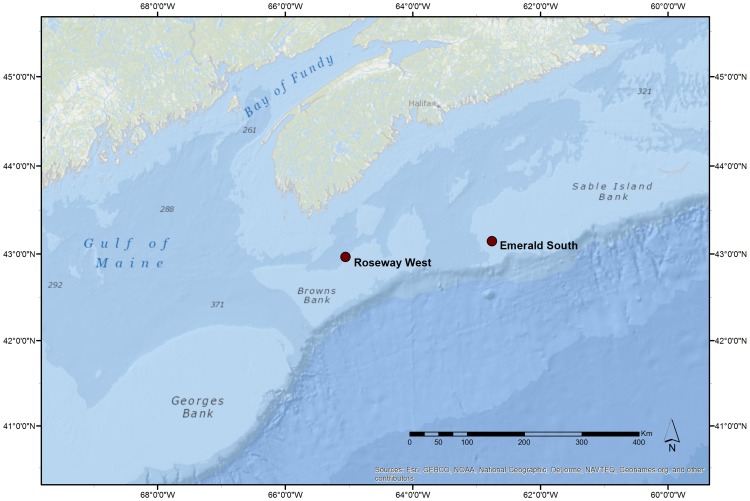
Locations of autonomous recording units on the Scotian Shelf. Roseway West: 42°58′00″ N 65°03′24″W; Emerald South: 43°8′54″N 62°45′42″W. Numbers correspond to water depth in meters.

Autonomous acoustic recording systems (NOAA/PMEL autonomous hydrophones, [32]) were deployed in two locations off the coast of Nova Scotia in June and July of 2004 (Figure 1) [5]. The recording systems recorded continuously for two years (July 2004–June 2006) at a sampling rate of 2 kHz, with a flat frequency response of ±3 dB from 30–840 Hz. This low frequency range includes the majority of right whale sound production, including the peak energy of right whale gunshot sounds [22], [33], [34]. Because water depth in the Scotian Shelf is less that 300 m, hydrophones were fixed 7 m above the sea floor to allow for optimum acoustic reception [5].

Recorders were positioned in two historical whaling areas in the North Atlantic [35]. One recording site, “Roseway West,” is a protected marine conservation area [36] and a known location for North Atlantic right whale surface-active groups [37]. The second recorder, “Emerald South,” was located approximately 200 km northeast of Roseway West on Emerald Bank.

### Seasonality Analysis

For each site, 24-hour periods were randomly selected from each week of data for the duration of the acoustic recordings. Spectrograms for each of these periods were visually inspected using RavenPro 1.5 [38] to detect gunshot presence in each hour (2 kHz sampling, Hann window, discrete Fourier transform (DFT) size 128, analysis resolution  = 15.6 Hz and 0.032 s, 50% overlap). The first 60 gunshots of a given hour were selected for subsequent diel analysis. Based on previous observations of gunshot bouts [26], a maximum of 60 gunshots was determined to be a reliable indicator of high gunshot activity. To test for seasonality, we determined the number of hours per day in which gunshots were detected. This did not take into consideration the total number of individual gunshots but rather assessed only the presence or absence of gunshot sounds in each hour (e.g. [6], [39]). The two sites, Roseway West and Emerald South, were analyzed separately.

### Diel Analysis

Diel patterns of right whale gunshot signals were also analyzed for both locations. For months of peak gunshot activity (August, September, October, November), each vocalization was coded based on light regime, and hourly averages (gunshots per hour) were calculated with a maximum of 60 gunshots per hour. The light code for each gunshot corresponded to one of four non-overlapping three-hour time blocks. These time blocks refer to sunrise/dawn, midday, sunset/dusk, and night, and were determined using data from the United States Naval Observatory [40].

### Statistics

For seasonal analysis, data from the two years of recordings were pooled and monthly averages of gunshot presence were calculated. These averages were compared via ANOVA to test for significant differences among months. To control for Type I error rate across multiple comparisons, Tukey's HSD was used as a one-step pairwise comparison procedure to test for differences between each of the months. For the diel analysis, the data were blocked by day and compared via ANOVA. Tukey's HSD was used to test for pairwise significance among the light regimes. All statistical tests were done using R v. 2.15.2 [41].

## Results

### Seasonality

**Figure 2:**
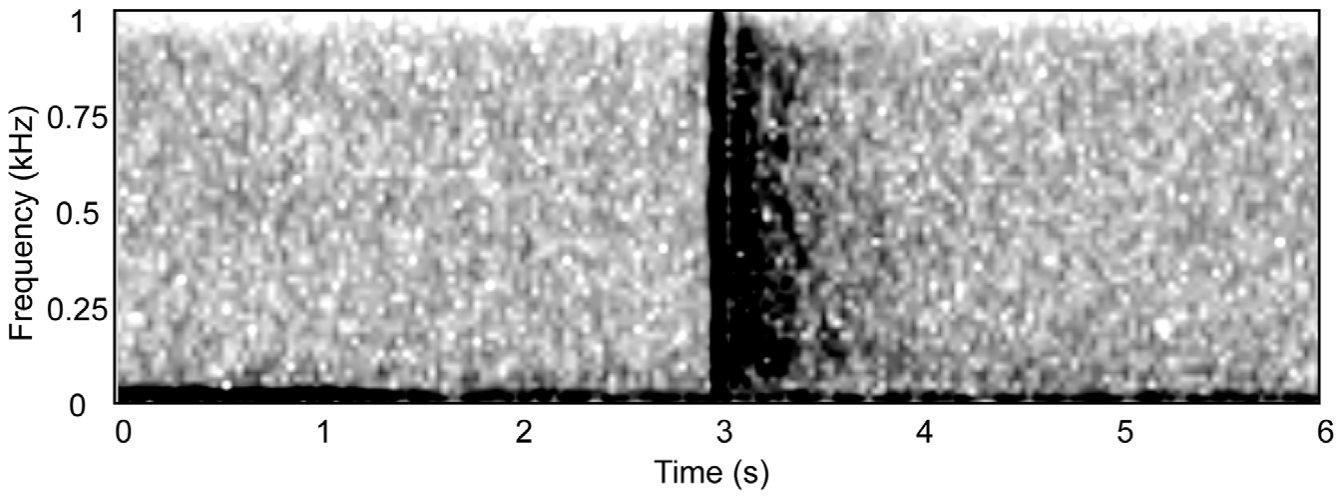
Spectrogram of a gunshot sound recorded in Roseway West. Spectrogram parameters: 2 kHz sampling, Hann window, discrete Fourier transform (DFT) size 128, analysis resolution  = 15.6 Hz and 0.032 s, 50% overlap.

**Figure 3:**
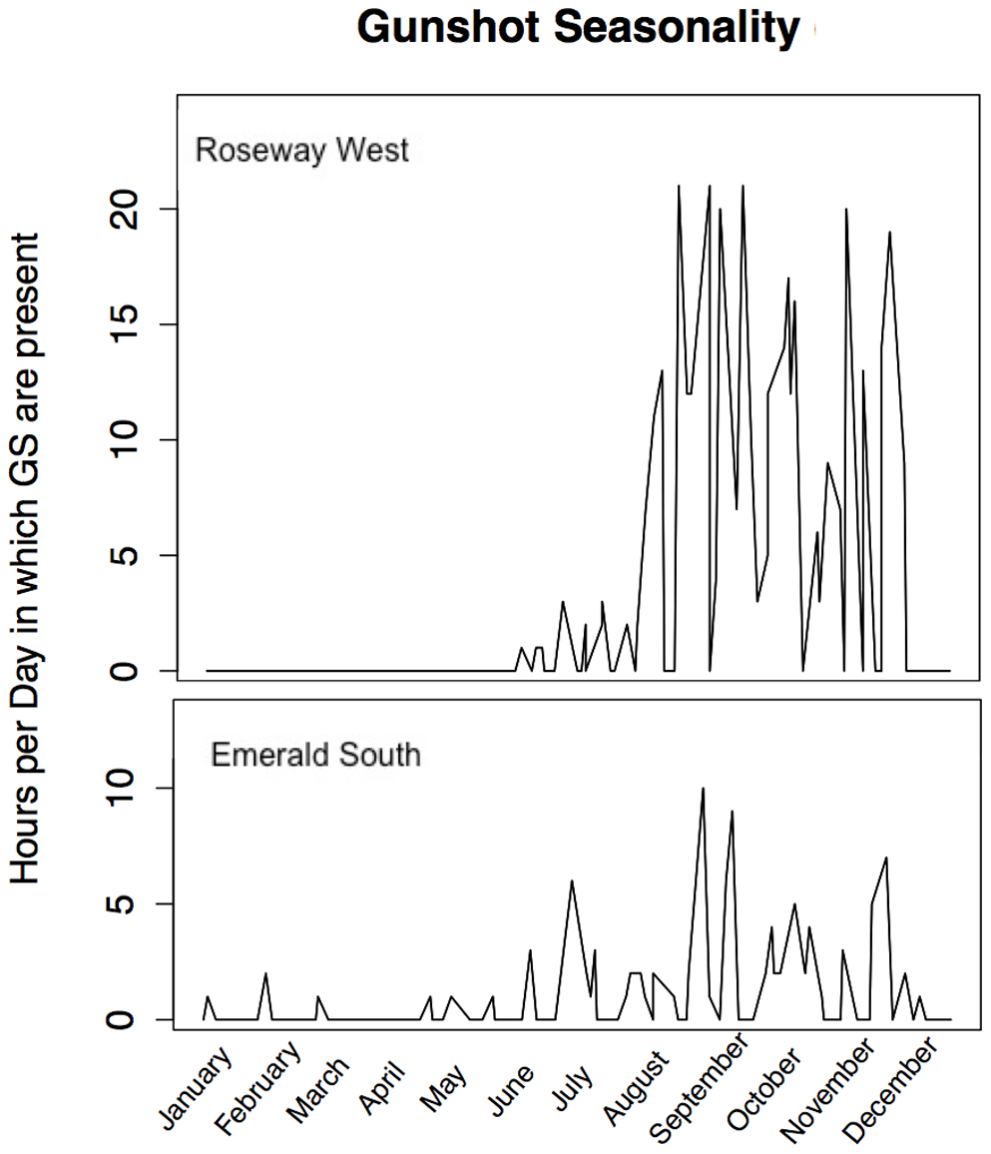
Seasonal trends of gunshot signal production. The hours per day with gunshots (GS) showed a seasonal pattern in both Roseway West (top) and Emerald South (bottom). In Roseway West, August-November had significantly more gunshot presence than other months. Emerald South, despite having fewer hours per day with gunshots overall, still exhibits a seasonal trend, with significantly more gunshot hours during the second half of the year.

Gunshot sounds (Figure 2) were detected seasonally in both sites in both years. The average and standard deviation of hours per day of gunshot activity were calculated for each site (Table 1). Gunshot activity was detected most of the year at Emerald South. Gunshots occurred in all months of the year at the Emerald location, while only occurring June through December at the Roseway West site. However, gunshot activity levels were higher in the Roseway West site, with more hours per day with gunshot presence detection. The peak month of gunshot activity for Roseway West was August (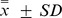: 10.78±7.61 hours/day), while the peak month of activity in Emerald South was October (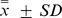: 2.20±1.69 hours/day). The maximum number of hours observed in a single day for Roseway West was 21 hours/day, while the maximum for Emerald South was 10 hours/day. ANOVA results (Table 2) for both sites indicated significance at α = 0.05. Further analysis via Tukey's HSD revealed that for Roseway West, the months of August, September, October, and November had a significantly higher number of hours of gunshot presence than other months. This indicates a strong seasonal trend in the Roseway site. The Emerald South site also showed a seasonal trend. Because this trend was weaker than Roseway, months were grouped into seasons (Jan-Mar, Apr-Jun, Jul-Sept, Oct-Dec) for pairwise analysis. Tukey's HSD indicated that the second half of the year (July-December) had a significantly higher number of hours of gunshots. Seasonal trends for both sites can be seen in Figure 3.

**Table 1:** Average and standard deviation of hours per day of gunshot activity for each site.

	Hours per Day in that Gunshots are Present ( 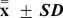 )											
	Jan	Feb	Mar	Apr	May	June	July	Aug	Sept	Oct	Nov	Dec
Roseway West	0.00±0.00	0.00±0.00	0.00±0.00	0.00±0.00	0.00±0.00	0.75±1.04	1.10±1.20	10.78±7.61	9.11±7.39	9.62±6.21	8.11±8.54	1.13±3.18
Emerald South	0.11±0.33	0.25±0.71	0.11±0.33	0.11±0.33	0.29±0.49	0.38±1.06	1.67±1.94	1.00±0.93	3.11±4.11	2.20±1.69	1.88±2.80	0.33±0.71

**Table 2:** ANOVA results for the seasonality analysis of both sites.

	Df	Sum Sq	Mean Sq	F Value	P
Roseway West	11	1952	177.48	8.949	1.14e-10
Residuals	93	1844	19.83		
Emerald South	11	101.1	9.193	3.089	0.000145
Residuals	91	270.8	2.976		

### Diel Trends

**Figure 4:**
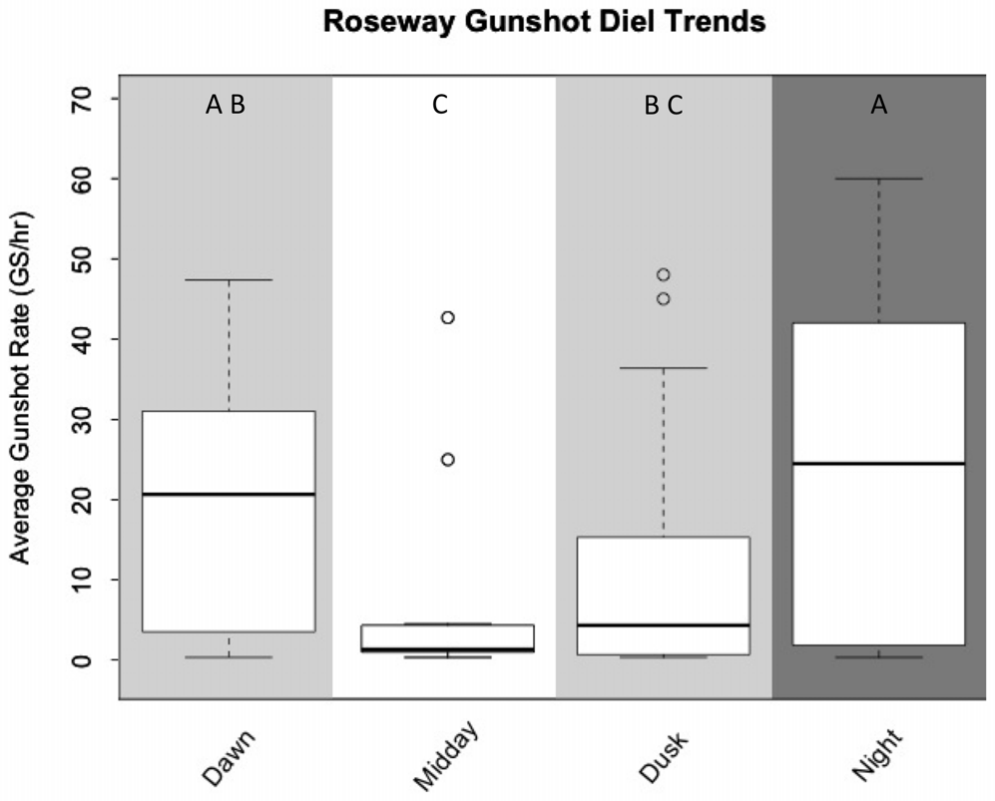
Gunshot diel trends in Roseway West. Gunshot rate (GS/hr) was significantly higher during night hours when compared to midday and dusk. There is a significant increase of gunshots from dusk to night and a clear decreasing trend of gunshot rate between night and dawn. Shading corresponds to light regime, and different letters indicate significant differences between them as determined by Tukey's Test.

Diel trends were analyzed for both sites. The average gunshot rates for each light regime (dawn, midday, dusk, night) for both sites can be seen in Table 3. ANOVA results (Table 4) revealed no significant diel trend for Emerald South (p = 0.551). There was, however, a trend for Roseway West (p = 1.83e-05). In Roseway West, gunshot activity peaked during the night hours (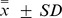: 24.30±21.67 GS/hr), and was lowest during midday (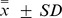: 6.78±12.62). Figure 4 demonstrates that gunshot rate increases from midday to night before tapering off during the dawn hours. Pairwise comparisons (Tukey's HSD) indicated significant differences in gunshot activity.

**Table 3:** Average and standard deviation of gunshot rates for the light regimes in both sites.

Site	Light Regime	Gunshot Rate (GS/hr; 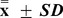 )
Roseway West	Midday	6.78±12.62
	Dusk	11.85±15.51
	Night	24.00±21.09
	Dawn	19.98±15.86
Emerald South	Midday	0.69±1.71
	Dusk	0.21±0.45
	Night	2.74±9.30
	Dawn	7.02±15.21

**Table 4:** ANOVA results for the diel analysis of both sites.

Site		Df	Sum Sq	Mean Sq	F Value	P
Roseway West	Day	29	14535	501.2	3.167	1.63e-05
	Light Regime	3	4597	1532.5	9.683	1.40e-05
	Residuals	87	13769	158.3		
Emerald South	Day	13	972	74.76	0.910	0.551
	Light Regime	3	405	134.91	1.642	0.195
	Residuals	39	3204	82.16		

## Discussion

Gunshot sound production is highly seasonal, with a significant increase in gunshot sound detection in the autumn. The timing of this increase is consistent with the current understanding that the right whale breeding season peaks in October-December [18], [29]–[31]. Detection of gunshot sounds can therefore be used as a reliable way to track the seasonal mating activities of right whales. Previously, the location of the majority of the right whale population during the breeding season was unknown [27], [42]. Data presented here indicate that the sites along the Scotian Shelf, in particular Roseway West, are potentially important locations for right whale breeding activities. Both seasonal and diel trends indicate variation in the usage of right whale gunshot sounds between the two sites. Roseway West has significantly more gunshot sounds overall, with a clear diel trend in production of these signals at night. Emerald South, while also showing seasonal trends in signal production, shows lower levels of gunshot sound production suggesting that either fewer whales utilize this habitat, or that the behavior of whales in this location varies from those on Roseway West.

### Seasonality

There was a similar seasonal trend at both recording sites, with the majority of gunshots occurring in August through November (Figure 3). This trend is consistent with the movement patterns of North Atlantic right whales, which move from the Bay of Fundy into areas off the Scotian Shelf during the middle to late summer [43], [44]. The gunshot seasonality observed here is also similar to the seasonal trend observed for right whale contact calls – i.e. upcalls – in these same locations in Roseway Basin [5].

Although right whale gunshot production in Emerald South exhibited seasonality, it was not as strong of a seasonal occurrence as observed at the Roseway West site. Previous studies [45] have used satellite tag data to indicate that right whale movements around the Scotian Shelf vary from year to year. This could be one possible explanation for the differences between the strength of the seasonal trend in Roseway West and Emerald South. It is also possible that differences in habitat use and prey abundance could account for the inter-site variation, with right whales preferring Roseway West to Emerald South because of denser prey aggregations [5].

There are three potential explanations for the seasonal pattern of gunshots. First, the increase in gunshot production could be directly tied to an increase in the number of whales at that location. The two recording sites analyzed here are located in areas historically associated with right whales [35], and the timing of high gunshot presence matches the annual movement patterns of right whales [44]. The movement of whales into and out of the Scotian Shelf could account for seasonal gunshot variation. Second, the seasonal increase in gunshots could be due to an increase in signal production by individual whales. This would indicate a shift in the behavior of right whales, possibly for reproductive purposes. Gunshots have only been documented in adult male North Atlantic right whales and this signal is thought to serve a reproductive function, whether that be as advertisement to females or as male-male agonistic displays [22]. An increase in gunshot production by individual males in the Scotian Shelf could indicate that right whales are using this area as a breeding ground, and the strong seasonal trend associated with Roseway West points to this location as a likely area for reproductive activity. A third explanation for gunshot seasonality is a combination of both increased whale density and increased acoustic signaling.

To better understand the reason behind the observed seasonality of gunshot sound production in these sites, it would be of interest to use passive acoustic monitoring to estimate North Atlantic right whale population density. These types of population estimations from passive acoustics have been done with other large cetaceans [46]–[48]. A more comprehensive knowledge of North Atlantic right whale population flux in a specific habitat would provide insight into the basis of observed seasonal acoustic trends.

### Diel Trends

Previous studies have indicated diel patterns in sound production in multiple cetacean species, including North Atlantic right whales [7], [8], [24], [49]–[51]. The data on North Atlantic right whales show results similar to those presented here for Roseway West, with increased calling activity at night and reduced calling activity during daytime hours. This periodicity may be due, in part, to prey abundance and distribution [7], [8]. North Atlantic right whales forage primarily on late-stage copepods, *Calanus finmarchicus* [52]. In Roseway Basin, these late-stage copepods aggregate in discrete layers [53]. It has been shown that in the summer feeding grounds (Bay of Fundy, Roseway Basin), North Atlantic right whales forage during the day when the prey is concentrated in deeper water [52]. The diel pattern of gunshot signaling in Roseway West supports the idea of an inverse relationship between calling and foraging. Similar diel trends in gunshot production have also been observed in the Bay of Fundy, another summer foraging location for North Atlantic right whales [26]. In both of these sites, aggregations of late-stage *C. finmarchicus* are likely composed of individuals in diapause [53]. It is possible that right whale individuals use visual cues to detect prey layers during foraging dives [52], thereby limiting efficient foraging to daytime hours. Increased levels of gunshot production during the nighttime hours allow individuals to signal without a great loss in foraging time.

Our results show that there is no significant diel trend for gunshots at Emerald South. It is possible that the differences between sites are due to food availability or differences in the demographics of the whales present at this site. There is an abundance of late-stage *C. finmarchicus* around Roseway West, while the Emerald South site is not in an area known for intensive right whale foraging [5]. While there was no significant diel trend for gunshots at this site, previous studies have shown diel trends in upcall production at Emerald South [5]. These results, however, show an opposite trend, with significantly more upcalls in the daytime hours. It has been hypothesized that, because of the low level of food availability in Emerald South, right whales spend more time socializing at this location [5].

## Conclusions

Remote acoustic monitoring is an important tool for understanding patterns in animal communication, and studies on the seasonality of context-specific acoustic signals allow inferences to be made about the behavior and habitat use of certain species. Our results further support the hypothesis that the North Atlantic right whale breeding season occurs in the later part of the year (August – November) and point to Roseway West as a potentially important breeding location for the North Atlantic right whale population. Future studies should focus on the behavioral context of gunshots on the Scotian Shelf and on the movements of right whales into and out of the recording locations. The hydrophone array used to collect this data allows for potential estimates of population size and individual calling rates [16]. Pairing population density measurements with the data presented here will allow a better understanding of right whale behavior and will be valuable information for future conservation efforts.
